# Research progress on small extracellular vesicles in diabetic nephropathy

**DOI:** 10.3389/fcell.2025.1535249

**Published:** 2025-03-05

**Authors:** Bingqing Yu, Lanfeng Wang, Yiping Mao, Xinyi Hu, Yukang Lu, Jiahui He, Xiaoying Yuan, Man Zhang, Zhiping Chen

**Affiliations:** ^1^ Department of Laboratory Medicine, First Affiliated Hospital of Gannan Medical University, Ganzhou, Jiangxi, China; ^2^ College of Medical Technology, Gannan Medical University, Ganzhou, Jiangxi, China; ^3^ Department of Nephrology, First Affiliated Hospital of Gannan Medical University, Ganzhou, Jiangxi, China; ^4^ First Clinical Medical College, Gannan Medical University, Ganzhou, Jiangxi, China

**Keywords:** diabetic nephropathy, small extracellular vesicles, therapy, biomarkers, progress

## Abstract

Virtually all cell types are capable of secreting small extracellular vesicles (sEV), which can be internalized by recipient cells, thereby serving as vehicles for intercellular communication. The cargoes of these vesicles, such as microRNAs, circular RNAs, proteins, and lipids, play significant roles in both normal cellular functions and the pathogenesis of various diseases. Diabetic Nephropathy (DN), a complication arising from diabetes, is expected to contribute to a 54% increase in the global diabetic population between 2015 and 2030, leading to substantial economic burdens on individuals and healthcare systems. sEVs, as promising biomarkers, demonstrate diverse mechanistic responses in different types of Diabetic Kidney Disease (DKD). They also hold advantages in the early prediction of renal damage. This article reviews the functional mechanisms of sEVs in DKD and their potential as therapeutic targets and biomarkers.

## 1 Introduction

DKD is the leading cause of CKD in many developed and developing regions (Ogurtsova et al., 2017). DN is the most common complication of diabetes, often culminating in end-stage renal disease (Sinha et al., 2020). In 2015, it was estimated that diabetes affected approximately 415 million individuals worldwide. Diabetes-related fatalities accounted for 5 million deaths, with the total global healthcare expenditure attributed to diabetes projected at $673 billion. By the year 2040, the prevalence of diabetes amongst individuals aged between 20 and 79 is anticipated to escalate to 642 million (Ogurtsova et al., 2017). DN manifests in approximately 20%–40% of patients diagnosed with diabetes (American Diabetes Association Professional Practice, 2022), nd represents the leading cause of end-stage kidney disease (ESKD) across diverse demographic groups (Selby and Taal, 2020). Type 2 diabetes comprises 90% of global diabetes cases, making it the predominant cause of DN. However, diagnosing DN is more complex in patients with type 2 diabetes in comparison to those with type 1 diabetes, often relying on the detection of retinopathy and the duration of disease onset. In cases where the diagnosis remains inconclusive, a kidney biopsy eventually serves as the definitive tool to confirm the presence of DN (Umanath and Lewis, 2018; Selby and Taal, 2020).

The International Society for Extracellular Vesicles (ISEV) defines “extracellular vesicles” (EVs) as lipid bilayer-bound particles that are incapable of self-replication (i.e., they lack a functional nucleus). These vesicles are released from cells and can originate from various sources, including bacteria, blood, urine, cerebrospinal fluid, saliva, synovial fluid, milk, and solid tissues. Based on their physical properties, the ISEV categorizes EVs into three main types: exosomes, microvesicles, and apoptotic bodies (Welsh et al., 2024). Exosomes are sEV released during cytokinesis from intracellular multivesicular bodies, which contain luminal vesicles formed by the double invagination of the plasma membrane (Kalluri and LeBleu, 2020). SEV are isolated using a variety of methods, with differential ultracentrifugation being the most commonly used and considered the gold standard technique (Sidhom et al., 2020). Researchers often employ a combination of separation techniques to enhance the yield and purity of the sEV obtained, but this approach can be both costly and time-consuming. Different types of EVs vary in size and characteristics (see Table 1). In general studies, to determine whether the extracted material is a sEV, we typically use a combination of transmission electron microscopy to identify sEV morphology, nanoparticle tracking analysis to identify sEV size, and Western blotting to identify protein markers on the surface of sEVs. This process is complex and time-consuming (Zhang et al., 2020).

**TABLE 1:** Classification, size, origin and surface molecules of EVs.

Type of EVs	Size	Surface molecules	Release	References
SEVs	<200 nm	CD63, CD81, CD9, Flotillin 1 ect	Endosomes-origin	Jia et al. (2022)
MVs	150–1,000 nm	Annexin A1, flotillin-2, selections, β1 integrins, annexin V, CD40, caveolin	Plasma membrane budding	Jeppesen et al. (2019), Zhou et al. (2019)
ApoBDs	50–1,000 nm	C1q, ICAM-3, CRT, CD44v6, PtdSer, oxidized low-density lipoprotein, and caspase-cleaved proteins	Released by dying or apoptotic cells	Zhou et al. (2022)

ApoBDs, apoptotic bodies; MVs, microvesicles; sEVs, small extracellular vesicles; C1q, c1q complement; ICAM-3, intercellular adhesion molecule 3; CRT, calreticulin; CD44v6, cell adhesion molecules CD44 variant 6; PtdSer, phosphatidylserine.

sEV are vesicular structures released from the parental cell into the extracellular fluid and subsequently taken up by recipient cells. They carry a diverse array of contents, including membrane proteins, cytoplasmic proteins, nuclear proteins, extracellular matrix proteins, metabolites, mRNA, non-coding RNA, circular RNA (circRNA), microRNA, and other substances. These vesicles play a role in intercellular communication, which may be involved in maintaining cellular homeostasis and regulating cellular functions (Meldolesi, 2018; Kalluri and LeBleu, 2020). Diabetic kidney injury begins with hyperglycemia, and a hyperglycemic environment damages nearly all kidney cell types, including glomerular podocytes, glomerular mesangial cells (GMC), glomerular endothelial cells (GEC), and tubular epithelial cells (TEC) (Kanwar et al., 2011). Under high glucose (HG) conditions, exocytosis from glomerular mesangial cells induces podocyte injury. The interaction of components from both cell types through sEV may trigger DN (Wang et al., 2018). It has been shown that diabetic patients develop diabetic cardiac fibrosis and diabetic cardiomyopathy through the mediation of sEVs delivered from cardiomyocytes to fibroblasts (Li et al., 2024). Additionally, the transfer of sEV enriched with arginase 1 to endothelial cells inhibits nitric oxide production, thereby leading to vascular dysfunction (Zhang et al., 2018). In addition, proteins and nucleic acids within sEVs can regulate or modify gene sequence information, thereby inducing heritable gene expression changes in target cells. These changes can manifest as DNA methylation, histone modification, and post-transcriptional regulation of RNA (Li et al., 2021; Ormazabal et al., 2022). Some researchers have found that circ_DLGAP4 within sEV promotes cell proliferation and fibrosis in patients with DN. This occurs by targeting downstream pathways through the sponge miR-143, thereby affecting gene expression products (Bai et al., 2020).

In the course of DKD, patients typically experience a progression from glomerular hyperfiltration to progressive albuminuria, a decline in Glomerular Filtration Rate, and ultimately ESRD. Previous reports have indicated that under HG conditions, the activation of the RhoA/ROCK pathway in GECs leads to increased endothelial permeability and dysfunction of the glomerular filtration barrier, subsequently resulting in glomerular damage and the development of albuminuria (Chen et al., 2021). The toxicity of persistent hyperglycemia not only generates more reactive oxygen species (ROS), inducing oxidative stress, but also leads to the deterioration of cellular or organ function through hyperglycemic stress or carbon stress pathways (Luo et al., 2015; Yan et al., 2016). Under conditions of hyperglycemia and oxidative stress, advanced glycation end-products (AGEs) are produced, leading to damage in various organs (Yamazaki et al., 2021). However, a new study has found that sEVs derived from human umbilical vein endothelial cells can protect against vascular calcification in diabetic patients when stimulated by AGEs (Guo et al., 2022). In addition, although overactivation of the renin-angiotensin-aldosterone system leads to increased proteinuria, proliferation of tethered cells, and the development of an inflammatory response, mouse studies have demonstrated that nanopreparations made by encapsulating angiotensin II blockers in sEV can effectively improve glucose tolerance and promote insulin synthesis and secretion (Singh et al., 2024). This type of nanotechnology, which delivers drugs by packaging them in sEV, can achieve the goal of delivering therapeutic drugs. However, the actual translation to the clinic with high carrier efficiency faces significant challenges (Meng et al., 2020).

## 2 Progress in diabetic nephropathy

### 2.1 Fibrosis

Renal fibrosis is a hallmark of advanced DN, resulting from long-term injury and dysregulation of the normal wound healing process, and is associated with excessive extracellular matrix deposition (Kanasaki et al., 2013). Podocytes play a crucial role in DN-related proteinuria as the final barrier to macromolecular influx into the urinary filtrate, and they become dysfunctional or depleted in this condition (Wu et al., 2017). Research has shown that under HG conditions, sEVs secreted by podocytes can induce and stimulate proximal tubular epithelial cells (PTECs). This leads to increased phosphorylation of p38 and Smad3, as well as enhanced expression of extracellular matrix proteins such as fibronectin (FN) and collagen IV (Col IV). These changes promote the deposition of the extracellular matrix and fibrosis in PTECs(Munkonda et al., 2018). In addition, Yang et al. found that sEV from macrophages treated with HG are taken up and internalized by renal tubular epithelial cells (RTECs). These HG-treated sEVs promote Col IV and plasminogen activator inhibitor-1 expression, and induce extracellular matrix (ECM) deposition in RTECs (Yang et al., 2023). Renal tubular epithelial cells and interstitial fibroblasts engage in intercellular communication, and to elucidate the mechanisms of this cellular crosstalk, Wen et al. co-cultured sEVs from high glucose-treated BUMPT cells, a renal tubular epithelial cell line, with NRK-49F cells, a renal fibroblast cell line. They observed that the fibroblasts showed a significant increase in the production of FN, α-SMA, and type I collagen, along with noticeable morphological changes. Despite a reduction in sEV secretion from renal tubular cells in patients with DKD, the sEVs produced by these cells exhibited potent pro-fibrotic activity on renal fibroblasts. Notably, the differential expression protein enolase 1 within the sEVs was associated with renal fibrosis in DKD (Wen et al., 2020).

In addition, research by Zhu et al. revealed that mesangial cells (MCs) stimulated with HG secrete sEVs that are enriched with circ_0125310. This circular RNA, circ_0125310, downregulates the expression level of miR-422a through direct interaction and an Argonaute 2-dependent mechanism. This downregulation relieves the inhibition on the expression of the insulin-like growth factor 1 receptor and p38, thereby promoting the proliferation and fibrosis of the mesangial cells (Zhu et al., 2021). Researchers Bai et al. have also revealed that sEVs from patients with DKD exhibit increased levels of circ_DLGAP4. This circular RNA acts as a molecular sponge for miR-143, indirectly regulating the ERBB3/NF-κB/MMP-2 axis. This regulatory action promotes fibrosis in MCs and, ultimately, facilitates the progression of DKD (Bai et al., 2020). There are also reports suggesting that sEVs derived from glucose-treated GECs can be internalized by GMCs. The circTAOK1 contained within these sEVs promotes proliferation, fibrosis, and Epithelial-Mesenchymal Transition (EMT) in GMCs by targeting the miR-520h/SMAD3 axis, providing new insights into the pathophysiological mechanisms of DKD (Li et al., 2022). In the investigative study by Liu et al., they reported elevated levels of miR-483-5p in both exosomes and urine derived from renal tissues of diabetic mice. Contrastingly, miR-483-5p was found at reduced levels in the TECs and renal tissues of these diabetic subjects. Furthermore, they demonstrated that the overexpression of miR-483-5p led to the suppression of renal interstitial fibrosis in the diabetic mouse model. This effect was attributed to the role of the transporter protein HNRNPA1, which facilitates the translocation of miR-483-5p from within TECs to the interstitial space of the tissue, as well as its excretion into urine via sEVs. Consequently, this translocation attenuated the binding of cellular miR-483-5p to the mRNAs of MAPK1 and TIMP2, culminating in enhanced progression of extracellular matrix deposition and the exacerbation of DN-induced renal interstitial fibrosis (Liu et al., 2021).

### 2.2 Transition

EMT and Endothelial-Mesenchymal Transition (EndMT) play crucial roles in the development of renal fibrosis. Both EMT and EndMT involve the loss of epithelial or endothelial cell properties and the acquisition of mesenchymal cell properties. Renal fibrosis induced by EMT and EndMT occurs when these transitions are stimulated by conditions such as inflammation or injury (Srivastava et al., 2013). Tsai et al. found that sEV derived from PTECs under HG conditions were enriched in the protein Fibulin-1 (FBLN1). The FBLN1 protein induced EMT by up-regulating the expression levels of N-cadherin and vimentin, and down-regulating the expression level of E-cadherin (Tsai et al., 2021). Research also demonstrates that in a HG state, sEVs derived from podocytes are rich in miR-221. After these sEVs are internalized by PTECs, their cargo, miR-221, mediates proximal tubular cell damage by targeting DKK2, which interacts with the Wnt/β-catenin signaling pathway. Importantly, through their analysis, they also found that the typical epithelial markers E-cadherin and ZO-1 were lost in expression, while the mesenchymal markers Vimentin and α-SMA were upregulated. This change in marker expression suggests a transition from an epithelial phenotype to a more mesenchymal phenotype (Su et al., 2020). In addition, GEC-derived sEVs (Ling et al., 2019), macrophage-derived sEVs (Yang et al., 2023), and sEVs isolated from human glomerular mesangial cells (HMCs) (Dong et al., 2022) have all been shown to promote EMT in the presence of HG.

Ning and colleagues discovered that sEVs derived from GECs treated under HG conditions contain elevated levels of miR-30a-5p. This miRNA modulates angiogenesis in GECs through the regulation of vascular endothelial growth factor (VEGF) via notch homolog protein 1 (Notch1). Additionally, they found that miR-30a-5p could potentially inhibit HG-induced EndMT in a Notch1-dependent manner (Ning et al., 2024). Although a number of studies have found that certain miRNAs are sufficient to induce the development of EMT or EndMT and promote the progression of DKD when sEVs secreted by one cell are internalized by another cell in the presence of HG, additional studies are still needed to elucidate the specific mechanisms involved.

### 2.3 Inflammation

Both systemic and renal-specific inflammation are pivotal in the onset and progression of DKD. Biopsies from patients with DN often reveal the infiltration of immune cells, particularly macrophages and lymphocytes. The infiltration and subsequent activation of these immune cells instigate the secretion of inflammatory and pro-fibrotic factors. In turn, these factors trigger an immune-inflammatory response that is instrumental in causing renal injury (Tang and Yiu, 2020). Previous reports have indicated that albumin stimulates the expression of interleukin-8 in PTECs, triggering intracellular signaling pathways associated with inflammation, which leads to glomerular damage (Tang et al., 2003).

In a study, Liu et al. discovered that sEVs from HK-2 cells treated with HG contained higher levels of Delta-like 4 (Dll4). The levels of Dll4 and Notch1 were positively correlated with the expression of Epsin1. With the progression of DN stages, the levels of Epsin1, Dll4, and Notch1 intracellular domain (N1ICD) in urinary sEVs from DN patients also increased gradually. They demonstrated that the protein Epsin1 regulates the release of Dll4 from TECs-derived sEVs. Dll4 is involved in the interaction between epithelial cells and macrophages, leading to the activation of N1ICD, an increase in the M1 macrophage phenotype, and the acceleration of inflammation under hyperglycemic conditions (Liu et al., 2023a). Zhu et al. found that sEVs derived from the Raw264.7 macrophage cell line treated with HG contain high levels of inducible nitric oxide synthase (iNOS) and interleukin-1β (IL-1β). iNOS and IL-1β trigger the production of pro-inflammatory and pro-fibrotic cytokines such as interleukin-6, tumor necrosis factor-α (TNF-α), monocyte chemoattractant protein-1, iNOS, α-SMA, and transforming growth factor-β1 through the activation of the NF-κB p65 signaling pathway. This promotes macrophage migration and activation, thereby causing kidney damage (Zhu et al., 2020). Other researchers, such as Liu and colleagues, observed significant macrophage infiltration in mice with DKD. They found that sEVs secreted by these macrophages are internalized by mesangial cells, leading to the downregulation of autophagy markers Beclin-1 and LC3B and the upregulation of the autophagy substrate p62 protein in mesangial cells, ultimately inhibiting autophagy in these cells. Importantly, they also discovered that sEVs promote the maturation of inflammatory cytokines by activating the NOD-like receptor 3 (NLRP3) inflammasome, thereby inducing inflammation. The NLRP3 inflammasome is a multiprotein complex that, upon activation, plays a critical role in the innate immune system by promoting the maturation and secretion of pro-inflammatory cytokines such as IL-1β and IL-18 (Liu et al., 2023b). Interestingly, Feng and colleagues observed that sEVs derived from patients with DN contain high levels of CCL21, and a significant infiltration of CD3 positive T cells (including both CD4 and CD8 positive T cell subsets) was noted in these patients. They also discovered a correlation between the accumulation of CD3 positive T cells and the levels of urinary sEVs. Therefore, CCL21-mediated T cell infiltration may be a key mechanism contributing to the chronic inflammation characteristic of DN (Feng et al., 2021).

In their investigative work, Lv et al. unveiled that in mouse models of acute or chronic kidney injury, sEVs derived from TECs are internalized by macrophages. These sEVs are rich in miR-19b-3p, which, at high levels, suppresses the expression of cytokine signaling inhibitor-1 in macrophages and upregulates the expression of inflammation-related factors. This leads to the activation of M1-type macrophages, promoting tubulointerstitial inflammation in the kidney. They also found similar phenomena in patients with diabetic kidney disease, where the content of TEC-sEVs-miR-19b-3p in urine was elevated and correlated with the severity of tubulointerstitial inflammation (Lv et al., 2019). Gao and colleagues discovered that miR-4449 is enriched in the serum sEVs of patients with DKD and that these serum sEVs are taken up by proximal renal epithelial cells. The sEVs regulate ROS levels, cell ferroptosis, and the expression of typical inflammatory cytokines through the miR-4449/HIC1 axis, thereby promoting the progression of inflammation in DKD (Gao et al., 2021).

**FIGURE 1:**
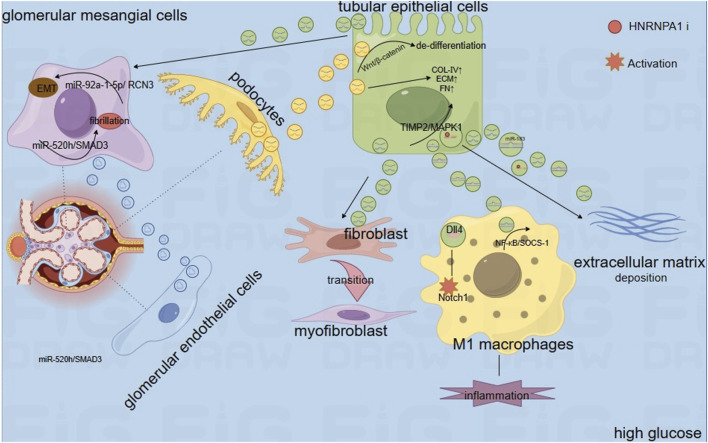
Therapeutic Role of Mesenchymal Stem Cell-Derived Small Extracellular Vesicles. Notes: Extracellular vesicles secreted by mesenchymal stem cells contain miR-146a-5p, miR-125a, miR-424-5p, and others. Among these, miR-146a-5p promotes the transformation of macrophages into M2 macrophages via the TRAF6/STAT1 axis. Similarly, macrophages overexpressing Sirt6 also foster the transformation into M2 macrophages. Furthermore, when small extracellular vesicles derived from M2 macrophages are internalized by podocytes, the miRNAs within these vesicles exert protective effects on the podocytes by targeting downstream pathways.

In summary, under HG conditions, sEVs derived from different cells promote the expression levels of TNF-α, IL-1β, and MCP-1 through various mechanisms by delivering their contents (proteins, microRNAs, and circRNAs), leading to inflammation. Additionally, they upregulate α-SMA, N-cadherin, vimentin, and FN, inducing EMT and EndMT, thereby promoting renal fibrosis. Figure 1 illustrates the development of inflammation and fibrosis induced by cargo transport between different cells via sEVs.

## 3 Treatment of diabetic nephropathy

### 3.1 Stem cell

Stem cell extracellular vesicle therapy has emerged as a promising breakthrough for the treatment of DN due to its self-renewal capabilities, multipotent differentiation potential, paracrine effects, and immunomodulatory properties. Compared to stem cells themselves, stem cell-derived sEV are likely to have lower immunogenicity, reduced toxicity, and an enhanced ability to cross biological barriers (Grange and Bussolati, 2022).

Mesenchymal stem cells (MSCs) derived from the umbilical cord, bone marrow, adipose tissue, and placenta possess the ability for self-renewal and differentiation into multiple cell lineages. MSC-derived small extracellular vesicles (MSC-sEVs) protect the kidneys by inhibiting apoptosis, necrosis, and oxidative stress in renal tubular epithelial cells, as well as suppressing harmful immune responses within the kidneys (Harrell et al., 2019). Xiang and colleagues’ research indicates that transplantation therapy using human umbilical cord mesenchymal stem cells (UC-MSCs) significantly reduces the levels of pro-inflammatory and pro-fibrotic factors in the kidneys and blood of DKD rats. This effectively improves renal function and inhibits and prevents inflammation and fibrosis. Moreover, they demonstrated through experiments that treating high-glucose-treated HK-2 cells with extracellular vesicles derived from human umbilical cord mesenchymal stem cells (UC-MSC-sEVs) also produced the same effects (Xiang et al., 2020). Similarly, Zhang and colleagues observed a significant elevation in the expression of inflammatory factors, such as IL-1β, in DKD rats. Treatment with UC-MSCs and UC-MSC-sEVs markedly reduced the expression of these inflammatory mediators. Furthermore, they demonstrated that UC-MSC-sEVs could ameliorate HG-induced EMT in tubular epithelial cells and reduce renal fibrosis in DN *in vitro* by targeting the Hedgehog/SMO signaling pathway (Zhang et al., 2024). In addition, Zhang and colleagues also demonstrated that there is a large amount of miR-146a-5p in exosomes derived from UC-MSCs. MiR-146a-5p promotes the polarization of M2 macrophages by inhibiting the Tumor Necrosis Factor Receptor-Associated Factor 6 (TRAF6)/Signal Transducer and Activator of Transcription 1 (STAT1) signaling pathway. M2 phenotype macrophages participate in immune regulation and promote tissue remodeling, thereby enhancing the protection against kidney. Furthermore, they found that the expression of Arginase 1 increased in DKD rats treated with UC-MSCs, further proving the transformation from M1 to M2 type macrophages (Zhang et al., 2022).

Hao and colleagues discovered that exosomes, which are derived from Adipose-Derived Mesenchymal Stem Cells (AD-MSCs), can ameliorate the symptoms of DN in experimental rat models. More specifically, the administration of these exosomes led to a notable decrease in parameters such as blood glucose levels, serum creatinine, 24-hour urinary protein excretion, Urine Albumin-to-Creatinine Ratio, and the ratio of kidney weight to body weight. Furthermore, these exosomes inhibited mesangial cell proliferation and renal fibrosis, both hallmarks of DN. Of note, the therapeutic exosomes from AD-MSCs were enriched with miR-125a. This microRNA directly interacts with Histone Deacetylase 1 (HDAC1), thereby preventing the deleterious effects of the interplay between HDAC1 and Endothelin-1 in the context of DN. This, in turn, mitigates the progression of DN (Hao et al., 2021). Cui and colleagues found that MSC-sEVs can alleviate the progression of DKD in db/db mice by reducing diabetic renal cell apoptosis and inhibiting the EMT. Treatment with MSCs or MSC-sEVs inhibits EMT in diabetic kidneys by upregulating E-cadherin expression and downregulating N-cadherin, α-SMA, and Snail (an EMT regulator) expression. Additionally, it upregulates the anti-apoptotic marker BCL2 and downregulates pro-apoptotic markers BAX and cleaved-caspase3 to exert anti-apoptotic effects. Moreover, miR-424-5p in MSC-sEVs targets Yes-associated protein 1 in HK-2 cells to inhibit HG-induced cell apoptosis and EMT (Cui et al., 2022). Additionally, Wang and colleagues demonstrated through experiments that DKD rats treated with bone marrow mesenchymal stem cell-derived small extracellular vesicles (BMMSC-sEVs) exhibited significant reductions in blood glucose, blood lipid, and blood viscosity levels compared to untreated DN mice. There was also a decrease in serum creatinine, blood urea nitrogen, and kidney injury molecule-1 levels, along with an increase in body weight and improved renal function. This improvement may be associated with the ability of BMMSC-sEVs to inhibit the activity of the JAK2/STAT3 signaling pathway (Wang et al., 2021).

Interestingly, new findings have emerged in urinary stem cells, with research indicating that exosomes derived from urinary stem cells are enriched with circRNA ATG7. This circRNA regulates the SOCS1/STAT3 signaling pathway by targeting miR-4500, leading to a downregulation of inflammatory mediators and an upregulation of IL-10 and Arg-1 expression levels. Consequently, this promotes the transformation of macrophage phenotypes from M1 to M2, thereby inhibiting the progression of DN (Sun et al., 2023). Jiang and colleagues discovered that sEV derived from urine-derived stem cell conditioned medium (USCs-sEVs) contain numerous potential factors, such as growth factors, transforming growth factor-β1, angiogenic factors, and bone morphogenetic protein-7. These factors may hold the potential to prevent diabetic kidney damage by inhibiting podocyte apoptosis and promoting vascular regeneration (Jiang et al., 2016). Additionally, research by Duan and colleagues has shown that human podocytes (HPDCs) internalize exosomes released by human urinary stem cells. These exosomes are rich in miR-16-5p, which can specifically bind to the 3′UTR (untranslated region) of VEGFA and downregulate its expression. The downregulation of VEGFA can in turn ameliorate podocyte injury caused by HG stimulation (Duan et al., 2019).

### 3.2 Macrophage

**FIGURE 2:**
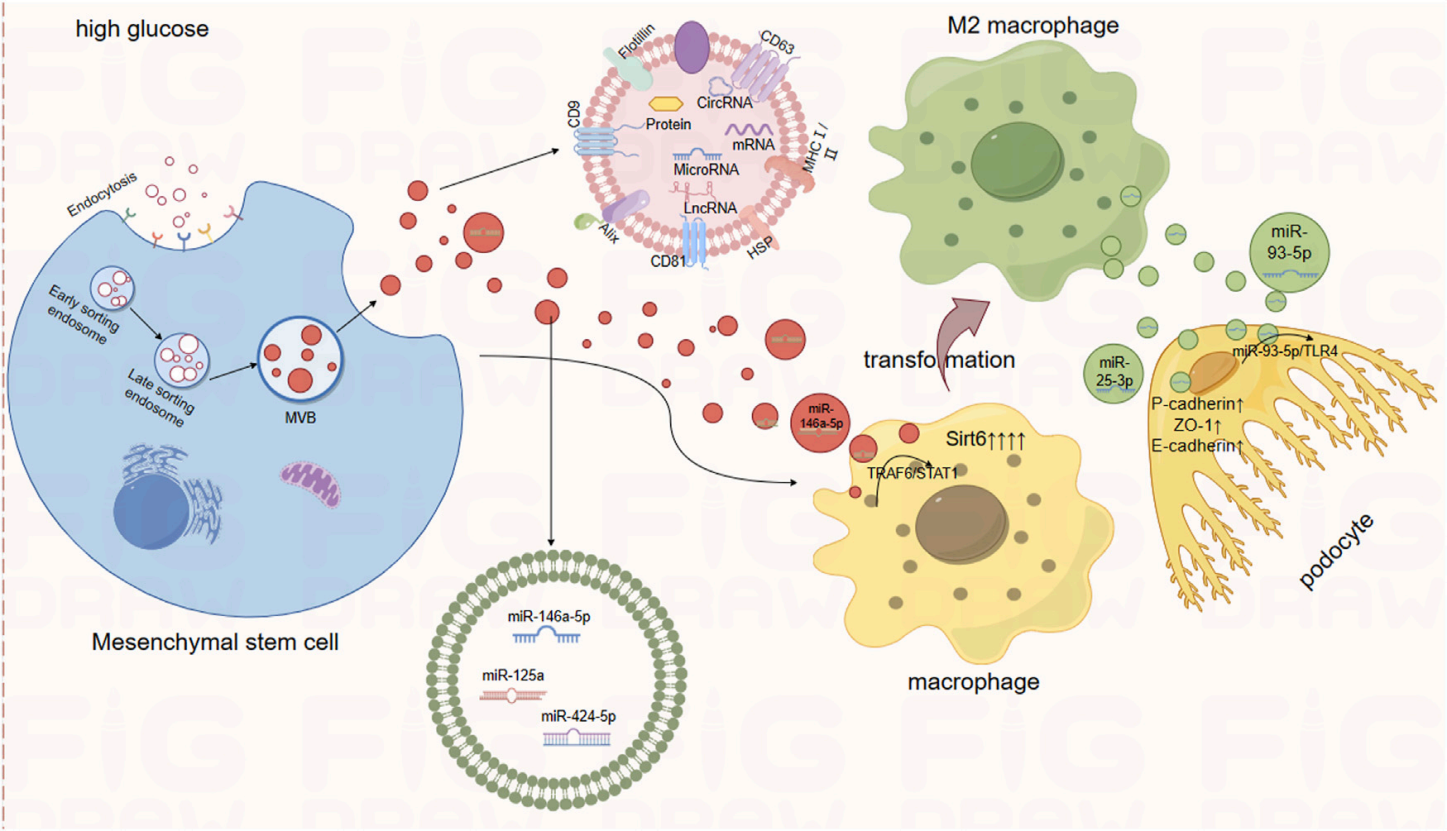
The Mechanism of Action of Extracellular Vesicles in DKD Through Intercellular Crosstalk. Notes: Extracellular vesicles influence the biological functions of recipient cells by transporting cargo between renal cells. This includes transfers from glomerular endothelial cells to mesangial cells, from tubular epithelial cells to mesangial cells, and from podocytes to tubular epithelial cells. Such intercellular exchanges ultimately lead to kidney damage, encompassing EMT, extracellular matrix deposition, inflammation, and fibrosis.

Similarly, in a study by Wang et al., it was discovered that sEV secreted by M2 macrophages attenuate lipopolysaccharide (LPS)-induced podocyte apoptosis by regulating the miR-93-5p/TLR4 axis (Wang et al., 2022). Additionally, the study found that sEVs from M2 macrophages are enriched with miR-25-3p. Once internalized by podocytes, miR-25-3p act on DUSP1, leading to the upregulation of P-cadherin, ZO-1, and E-cadherin, thereby alleviating HG-induced podocyte damage. Podocyte injury is crucial in the progression of DN, and exploring the mechanisms of podocyte damage in DN may lead to future therapeutic strategies for the condition (Huang et al., 2020) (Figure 2).

### 3.3 Milk

Recently, a new nano drug delivery technology has been reported, in which milk-derived exosomes are used for the oral administration of the chemotherapy drug paclitaxel (Agrawal et al., 2017). Compared to sEVs from other sources, milk-derived exosomes exhibit several advantages. As an ideal oral drug delivery vehicle, milk-derived sEVs offer excellent biocompatibility, stability, and bioavailability (Meng et al., 2020). However, there is currently limited research on this topic in the context of DKD. Shaban et al. demonstrated that treatment with camel milk and/or its exosomes in a streptozotocin (STZ)-induced DN rat model alleviates renal injury parameters, lowers blood glucose levels, increases antioxidant enzymes (such as SOD, CAT, and GPx), and reduces the expression levels of the lipid peroxidation biomarker MDA, thereby restoring oxidative stress associated with DN. Additionally, the expression of fibrosis-related genes (TGFβ1 and ICAM1), kidney injury markers, and matrix remodeling-related genes (ETS1, ITGβ2, and TIMP2) were significantly reduced, with the lowest expression levels observed in the combined treatment group. This study provides evidence that camel milk and its sEVs can mitigate the harmful effects of DN (Shaban et al., 2022).

### 3.4 Engineered small extracellular vesicles

Leveraging the properties of sEVs, engineered sEVs can be designed to serve as drug delivery systems for immunotherapy. ^55^For instance, various biological modifications can be made to the membranes of sEVs to endow them with specific characteristics (Zhang et al., 2023). In research conducted by Ji and colleagues, they propose that rvg -miR-23a/27a/26a-Exos could be used as a nanotherapeutic drug for the treatment of DKD. They discovered that the engineered RVG modified exosomes (RVG-cluster 3-Exos) containing miR-23a-3p, miR-26a-5p, and miR27a-3p significantly inhibited the synthesis of fibrosis-associated proteins (such as α-SMA, collagen I, and fibronectin), and increased the expression of E-cadherin in TECs. These changes help to ameliorate the progression of ECM deposition mediated by TECs.Furthermore, the miR-23a/27a/26a cluster targets Lipoma Preferred Partner, which can improve the progression of tubulointerstitial fibrosis in DN. It also targets Zbtb20 and Klhl42 to enhance the ability to improve fibrosis (Ji et al., 2023). These engineered small extracellular vesicles (sEVs) can also be referred to as exogenous loading. They can be modified through methods such as electroporation or heat shock after isolation (de Abreu et al., 2021). Depending on the timing of the intervention, there is also endogenous loading of sEVs, which involves genetically modifying the cells that secrete sEVs to indirectly influence the cargo they carry. Both loading techniques have their advantages and disadvantages. Due to the immaturity and instability of current sEVs isolation techniques, it is challenging to transition from laboratory research to clinical applications (Richards et al., 2023).

### 3.5 Others

Drugs acting through sEVs can significantly improve renal function and proteinuria in DN mice, and reduce the glomerular volume and mesangial matrix expansion in DN mice. Liang et al. found that Yi-shen-hua-shi (YSHS) granules might improve podocyte injury and inhibit the progression of DKD by inhibiting M1 macrophage polarization and reducing the miRNA content in macrophage-derived exosomes (Liang et al., 2022). In other studies on DKD, researchers such as Mishra et al. found that autologous reinfusion of sEVs isolated from the urine of DN mice effectively reduced inflammatory factors and fibrosis markers in the kidneys. This effect may be related to the excretion of protective miRNAs through sEVs into the urine, as these protective miRNAs are downregulated in DKD, leading to the upregulation of their target genes such as AKT3 and FOXO4, ultimately contributing to the progression of DN (Mishra et al., 2023).

In summary, sEVs derived mainly from stem cells and macrophages exert inhibitory effects on the progression of DKD through various mechanisms. Recent articles have reported that exosomes have therapeutic potential in various conditions such as scar acne (Kwon et al., 2020), chronic wounds (Johnson et al., 2023), skin aging (Park et al., 2023), and spinal cord injury (Akhlaghpasand et al., 2024). Nunez Lopez et al. demonstrated through clinical trials that pioglitazone can improve insulin sensitivity and glucose homeostasis in patients with type 2 diabetes by modulating the levels of miRNAs within sEV derived from adipose tissue (Nunez Lopez et al., 2022). In recent years, there have been few clinical studies related to sEV in DN. However, to achieve the practical clinical application of drugs mediated by sEV to improve the disease, many challenges remain. One of the main challenges is the efficiency and quality of sEVs extraction. Whether using two-dimensional or three-dimensional EV production culture systems, no current technology can achieve large-scale production and cost-effective EV acquisition (Patel et al., 2018). In the future, we should focus on creating large-scale manufacturing platforms and technologies to address the first step in applying sEVs. Additionally, the contents of sEVs from different sources can vary, and even sEVs from the same type of cell can exhibit compositional heterogeneity. This heterogeneity poses a significant obstacle to the translation of sEVs into clinical trials.

## 4 Diagnostic markers

The commonly used biomarkers for DKD include GFR, albuminuria, creatinine, and cystatin C, among others. Each of these biomarkers has its own advantages and disadvantages. Among them, the GFR tracked after diagnosis is the best predictor of future ESRD; albuminuria is used to predict the progression of DKD, but it lacks specificity and sensitivity for the progression to ESRD and the decline in eGFR (estimated GFR) (Colhoun and Marcovecchio, 2018). The most studied biofluid for EVs is blood, followed by urine. Compared to blood, urine has the advantage of being non-invasively, continuously, and abundantly obtainable. However, urine-derived EVs originate from multiple parts of the urogenital tract, such as the kidneys, bladder, prostate (in males), and uterus-vagina (in females). This makes it more challenging to interpret the source of urinary sEVs and their potential significance in disease processes. Despite these challenges, urinary sEVs hold great promise as potential biomarkers for kidney diseases, given that they can reflect the ongoing pathological processes in the kidneys (Erdbrügger et al., 2021).

For sEVs derived from renal tubular epithelial cells stimulated with HG, there are significant differences in the expression of lncRNAs, mRNAs, circRNAs, miRNAs, proteins, and lipids compared to normal controls. These differentially expressed RNAs are involved in the progression of DN and may serve as biomarkers for the disease (Zhou et al., 2021). In the study of DN, normal mice are typically induced to become diabetic mice to observe changes in various indicators, including the changes in target indicators. For instance, the work by Mohan et al. involved the induction of diabetes mellitus (DM) in male Wistar rats through a single intraperitoneal administration of streptozotocin. This led to a notable increase in both the tubulointerstitial fibrosis index and glomerulosclerosis index in the renal tissues of the diabetic rats relative to non-diabetic controls and insulin-treated diabetic cohorts (DM + INS). Furthermore, microRNA miR-451-5p levels were significantly upregulated in the urinary exosomes of the untreated diabetic rats (DM), particularly between the 3- and 6-week marks. Notably, the presence of UE miR-451-5p at 6 weeks served as a predictive biomarker for urinary albumin levels at 9 weeks. Thus, urinary sEVs miR-451-5p emerges as a potential early detection biomarker for renal injury in diabetic kidney disease (Sands et al., 2016).

To investigate clinically significant biomarkers in DN, many researchers conduct experiments using clinical samples. In the realm of DKD research, Feng et al. made a significant observation that patients with DN exhibited a higher excretion of sEVs in their urine compared to patients with DM and healthy individuals. Notably, the mRNA of chemokine (C-C motif) ligand 21 (CCL21), encapsulated within these urinary sEVs, was found to be elevated in the DN cohort. This increase in CCL21 mRNA levels showed a strong positive correlation with the severity of 24-h proteinuria and an inverse relationship with the eGFR. These findings suggest that CCL21 mRNA in urinary sEVs could potentially serve as a more effective biomarker for the early detection of DN in patients with type 2 diabetes mellitus (T2DM), offering advantages over traditional measures such as eGFR and proteinuria levels (Feng et al., 2021). In a study conducted by Pan et al., certain metabolites encapsulated within sEVs—namely uracil, 4-acetylaminobutyric acid, sphingosine 1-phosphate, and lysophosphatidylcholine—were identified as potential biomarkers for the early detection of DKD. The combined analysis of these four candidate metabolites could potentially improve the predictive accuracy for DKD (Pan et al., 2022).

In one study, large-scale proteomic analysis of urine and urinary sEVs was conducted on T2DM patients with and without DKD at different stages. The analysis revealed differential protein expression across various stages of DKD. Specifically, the levels of five proteins—albumin, IGHG2, TF, SERPINA1, and MYO5A—increased significantly as DKD progressed (Du et al., 2023). Additionally, other studies have shown that CD133+ uEVs can reflect glomerular conditions. Researchers found that the levels of CD133 within extracellular vesicles released into urine (uEVs) significantly decrease in the presence of glomerular damage (Dimuccio et al., 2020).

The molecular cargo of sEVs, shielded by their phospholipid bilayer membrane, is safeguarded against degradation and environmental fluctuations, thereby providing a more accurate reflection of the miRNA, mRNA, lncRNA, and protein profile alterations emanating from renal pathologies. Notably, renal structural changes in some diabetic patients precede the onset of proteinuria, underscoring the imperative need for novel biomarkers. An increasing body of evidence supports the potential of sEVs contents as biomarkers for DKD. The identification of these biomolecular changes in sEVs could bridge the current diagnostic gap, offering a promising avenue for early detection of DKD. In addition, there are other biomarkers exemplified in the Table 2 below.

**TABLE 2:** sEVs as biomarkers of DKD and preclinical evidence.

sEVs	Type of DKD	Marker	Number of DKD patients	Patients	Expression	References
sEVs- proteins	DN	PHYHD1	12	DN VS NDRD VS HC VS T2DM	Upregulated	Ding et al. (2023)
	DN	CALM1	44	T2DM VS DKD VS HC	Upregulated	Li et al. (2023)
	DN	PAK6	48	HC VS T2DM VS DN	Upregulated	Sinha et al. (2023)
		EGFR			Upregulated	
sEVs- Mrna	DN	WT1 mRNA	103	DM VS DN VS HC	Upregulated	Hashemi et al. (2021)
		ACE mRNA				
	DN	CDH2, MCP-1 mRNA	88	DM VS DN VS HC	Downregulated	Abdel Ghafar et al. (2022)
sEVs-micro-RNA	Early T1DN	miR-145	12	ND VS T1DM VS T1DN	Upregulated	Martelli et al. (2013)
	T2DN	miR-4534	17	T2DM VS T2DN	Upregulated	Zhao et al. (2020)
	T2DN	miR-877-3p	5	T2DM VS HC VS T2DN	Upregulated	Xie et al. (2017)
		miR-150-5p			Upregulated	
		miR-15a-5p			Downregulated	
	T2DN	miR-320c	8	HC VS T2DM VS DN	Upregulated	Jeyaseelan et al. (2016)
		miR-6068				
	T2DN	miR-15b, miR-34a; miR-636	54	ND VS PDKD VS NPDKD	Upregulated	Eissa et al. (2016a)
	T2DN	let-7c-5p	28	HC VS T2DN VS T2DM	Upregulated	Li et al. (2018)
	T2DN	miR-133b	110	T2DN VS HC VS T2DM	Upregulated	Eissa et al. (2016b)
		miR-342				
		miR-30a				
	DN	miR-615-3p	42	DKD VS HC VS T2DM	Upregulated	Wang et al. (2023)
	DN	miR-663a	9	PDKD VS NPDKD VS HC VS T2DM	Downregulated	Sinha et al. (2023)
	DN	miR-188-5p	6	non-diabetic CKD VS DKD	Upregulated	Lee et al. (2020)
		miR-150-3p				
		miR-133a-3p			Downregulated	
		miR-153-3p				
	DN	miR-145-5p	20	T2DM VS DN VS NC	Upregulated	Han et al. (2023)
	DN	miR-4449	23	HV VS DM VS DN	Upregulated	Kim et al. (2019)
	DN	miR-126	46	NAlb DN VS MiAlb DN VS MaAlb	Upregulated	Dimuccio et al. (2022)
		miR-145				
	T2DN	miR-30b-5p	14	T2DNRF VS T2DKD VS T2DM	Downregulated	Zang et al. (2019)
		miR-21-5p			Upregulated	

NDRD, non-diabetic renal disease; PHYHD1, phytanoyl-CoA dioxygenase domain containing 1; T2DM, Type 2 diabetes mellitus; NPDKD, non-proteinuric DKD; T2DNRF, type 2 diabetes mellitus and normal renal function; HV, healthy volunteers; NAlb DN, normoalbuminuria; MiAlb DN, microalbuminuria; MaAlb DN, macroalbuminuria.

T2DN, type 2 diabetic nephropathy; T1DN, type 1 diabetic nephropathy; HC, healthy controls; CALM1, Calmodulin-1; ND, non-diabetic subjects; PDKD, proteinuric diabetic kidney disease.

## 5 Conclusion and future perspectives

With the rise in living standards, the consumption of high-sugar and high-fat foods has contributed to the growing prevalence of DN. This condition manifests in individuals with diabetes as glomerular hypertrophy, glomerulosclerosis, and tubulointerstitial inflammation and fibrosis. Despite the availability of therapeutic interventions, managing the complications associated with diabetes remains a formidable challenge. There is a pressing need to identify novel strategies and tools for the effective treatment of DN. sEVs have emerged as key mediators of intercellular communication, influencing the biological functions of target cells and offering new insights into the pathogenesis and potential therapeutic avenues for DKD.

sEVs have a nanoscale size, low immunogenicity, and the ability to cross the blood-brain barrier, making them an emerging tool in delivery systems in recent years. However, there are still numerous challenges to be addressed before sEVs can be effectively used in clinical therapy and as diagnostic biomarkers. Firstly, the isolation of sEVs is a major challenge. Current isolation methods cannot achieve high-purity sEVs samples, often resulting in the presence of non-sEVs components. Additionally, there is no consensus on the characterization of sEVs, making it difficult to obtain highly pure and specific sEVs subgroups. This poses a problem for targeted delivery to recipient cells. Furthermore, sEVs are rapidly cleared by the liver or kidneys when circulating in the system, necessitating the use of high concentrations of nanocarriers for effective delivery. The stability of sEVs is another concern; they require appropriate preservation methods after extraction to maintain their integrity. To bring therapeutic sEVs into clinical practice, collaboration, guidance, and oversight from regulatory bodies like the Food and Drug Administration (FDA) are essential to ensure systematic, standardized, and legal processes. Therefore, the entire process of obtaining, processing, and preserving sEVs needs to be standardized and unified to overcome these challenges.

## References

[B1] Abdel Ghafar M. T. Dehghanbanadaki H. Forouzanfar K. Kakaei A. Zeidi S. Salehi N. (2022). The role of CDH2 and MCP-1 mRNAs of blood extracellular vesicles in predicting early-stage diabetic nephropathy. Plos One 17 (4), e0265619. 10.1371/journal.pone.0265619 35363774 PMC8975111

[B2] Agrawal A. K. Aqil F. Jeyabalan J. Spencer W. A. Beck J. Gachuki B. W. (2017). Milk-derived exosomes for oral delivery of paclitaxel. Nanomedicine 13 (5), 1627–1636. 10.1016/j.nano.2017.03.001 28300659

[B3] Akhlaghpasand M. Tavanaei R. Hosseinpoor M. Yazdani K. O. Soleimani A. Zoshk M. Y. (2024). Safety and potential effects of intrathecal injection of allogeneic human umbilical cord mesenchymal stem cell-derived exosomes in complete subacute spinal cord injury: a first-in-human, single-arm, open-label, phase I clinical trial. Stem Cell Res. & Ther. 15 (1), 264. 10.1186/s13287-024-03868-0 39183334 PMC11346059

[B4] American Diabetes Association Professional Practice, C. (2022). Chronic kidney disease and risk management: standards of medical care in diabetes-2022. Diabetes Care 45, S175–S184. 10.2337/dc22-S011 34964873

[B5] Bai S. Xiong X. Tang B. Ji T. Li X. Qu X. (2020). Exosomal circ_DLGAP4 promotes diabetic kidney disease progression by sponging miR-143 and targeting ERBB3/NF-κB/MMP-2 axis. Cell Death & Dis. 11 (11), 1008. 10.1038/s41419-020-03169-3 PMC7683700 33230102

[B6] Chen X. Chen J. Li X. Yu Z. (2021). Activation of mTOR mediates hyperglycemia-induced renal glomerular endothelial hyperpermeability via the RhoA/ROCK/pMLC signaling pathway. Diabetology & Metabolic Syndrome 13 (1), 105. 10.1186/s13098-021-00723-7 34627341 PMC8501565

[B7] Colhoun H. M. Marcovecchio M. L. (2018). Biomarkers of diabetic kidney disease. Diabetologia 61 (5), 996–1011. 10.1007/s00125-018-4567-5 29520581 PMC6448994

[B8] Cui C. Zang N. Song J. Guo X. He Q. Hu H. (2022). Exosomes derived from mesenchymal stem cells attenuate diabetic kidney disease by inhibiting cell apoptosis and epithelial‐to‐mesenchymal transition via miR‐424‐5p. FASEB J. 36 (10), e22517. 10.1096/fj.202200488R 36036527

[B9] de Abreu R. C. Ramos C. V. Becher C. Lino M. Jesus C. da Costa Martins P. A. (2021). Exogenous loading of miRNAs into small extracellular vesicles. J. Extracell. Vesicles 10 (10), e12111. 10.1002/jev2.12111 34377372 PMC8329988

[B10] Dimuccio V. Peruzzi L. Brizzi M. F. Cocchi E. Fop F. Boido A. (2020). Acute and chronic glomerular damage is associated with reduced CD133 expression in urinary extracellular vesicles. Am. J. Physiology-Renal Physiology 318 (2), F486–F495. 10.1152/ajprenal.00404.2019 31869243

[B11] Dimuccio V. Bellucci L. Genta M. Grange C. Brizzi M. F. Gili M. (2022). Upregulation of miR145 and miR126 in EVs from renal cells undergoing EMT and urine of diabetic nephropathy patients. Int. J. Mol. Sci. 23 (20), 12098. 10.3390/ijms232012098 36292960 PMC9603196

[B12] Ding X. Zhang D. Ren Q. Hu Y. Wang J. Hao J. (2023). Identification of a non-invasive urinary exosomal biomarker for diabetic nephropathy using data-independent acquisition proteomics. Int. J. Mol. Sci. 24 (17), 13560. 10.3390/ijms241713560 37686366 PMC10488032

[B13] Dong Q. Dong L. Zhu Y. Wang X. Li Z. Zhang L. (2022). Circular ribonucleic acid nucleoporin 98 knockdown alleviates high glucose-induced proliferation, fibrosis, inflammation and oxidative stress in human glomerular mesangial cells by regulating the microribonucleic acid-151-3p-high mobility group AT-hook 2 axis. J. Diabetes Investigation 13 (8), 1303–1315. 10.1111/jdi.13821 PMC9340880 35482475

[B14] Du S. Zhai L. Ye S. Wang L. Liu M. Tan M. (2023). In-depth urinary and exosome proteome profiling analysis identifies novel biomarkers for diabetic kidney disease. Sci. China Life Sci. 66 (11), 2587–2603. 10.1007/s11427-022-2348-0 37405567

[B15] Duan Y. R. Chen B. P. Chen F. Yang S. X. Zhu C. Y. Ma Y. L. (2019). Exosomal microRNA‐16‐5p from human urine‐derived stem cells ameliorates diabetic nephropathy through protection of podocyte. J. Cell. Mol. Med. 25 (23), 10798–10813. 10.1111/jcmm.14558 31568645 PMC8642687

[B16] Eissa S. Matboli M. Aboushahba R. Bekhet M. M. Soliman Y. (2016a). Urinary exosomal microRNA panel unravels novel biomarkers for diagnosis of type 2 diabetic kidney disease. J. Diabetes its Complicat. 30 (8), 1585–1592. 10.1016/j.jdiacomp.2016.07.012 27475263

[B17] Eissa S. Matboli M. Bekhet M. M. (2016b). Clinical verification of a novel urinary microRNA panal: 133b, -342 and -30 as biomarkers for diabetic nephropathy identified by bioinformatics analysis. Biomed. & Pharmacother. 83, 92–99. 10.1016/j.biopha.2016.06.018 27470555

[B18] Erdbrügger U. Blijdorp C. J. Bijnsdorp I. V. Borràs F. E. Burger D. Bussolati B. (2021). Urinary extracellular vesicles: a position paper by the urine task force of the international society for extracellular vesicles. J. Extracell. Vesicles 10 (7), e12093. 10.1002/jev2.12093 34035881 PMC8138533

[B19] Feng Y. Zhong X. Ni H.-F. Wang C. Tang T.-T. Wang L.-T. (2021). Urinary small extracellular vesicles derived CCL21 mRNA as biomarker linked with pathogenesis for diabetic nephropathy. J. Transl. Med. 19 (1), 355. 10.1186/s12967-021-03030-x 34404433 PMC8371892

[B20] Gao C. Wang B. Chen Q. Wang M. Fei X. Zhao N. (2021). Serum exosomes from diabetic kidney disease patients promote pyroptosis and oxidative stress through the miR-4449/HIC1 pathway. Nutr. & Diabetes 11 (1), 33. 10.1038/s41387-021-00175-y PMC8566490 34732690

[B21] Grange C. Bussolati B. (2022). Extracellular vesicles in kidney disease. Nat. Rev. Nephrol. 18 (8), 499–513. 10.1038/s41581-022-00586-9 35641620 PMC9152665

[B22] Guo B. Shan S.-K. Xu F. Lin X. Li F.-X.-z. Wang Y. (2022). Protective role of small extracellular vesicles derived from HUVECs treated with AGEs in diabetic vascular calcification. J. Nanobiotechnology 20 (1), 334. 10.1186/s12951-022-01529-z 35842695 PMC9287893

[B23] Han L. Wang S. Li J. Zhao L. Zhou H. (2023). Urinary exosomes from patients with diabetic kidney disease induced podocyte apoptosis via microRNA-145-5p/Srgap2 and the RhoA/ROCK pathway. Exp. Mol. Pathology 134, 104877. 10.1016/j.yexmp.2023.104877 37952894

[B24] Hao Y. Miao J. Liu W. Cai K. Huang X. Peng L. (2021). Mesenchymal stem cell-derived exosomes carry MicroRNA-125a to protect against diabetic nephropathy by targeting histone Deacetylase 1 and downregulating endothelin-1. Diabetes, Metabolic Syndrome Obes. Targets Ther. 14, 1405–1418. 10.2147/dmso.S286191 PMC8006976 33790607

[B25] Harrell C. R. Jovicic N. Djonov V. Arsenijevic N. Volarevic V. (2019). Mesenchymal stem cell-derived exosomes and other extracellular vesicles as new remedies in the therapy of inflammatory diseases. Cells 8 (12), 1605. 10.3390/cells8121605 31835680 PMC6952783

[B26] Hashemi E. Dehghanbanadaki H. Baharanchi A. A. Forouzanfar K. Kakaei A. Mohammadi S. M. (2021). WT1 and ACE mRNAs of blood extracellular vesicle as biomarkers of diabetic nephropathy. J. Transl. Med. 19 (1), 299. 10.1186/s12967-021-02964-6 34246281 PMC8272332

[B27] Huang H. Liu H. Tang J. Xu W. Gan H. Fan Q. (2020). M2 macrophage‐derived exosomal miR‐25‐3p improves high glucose‐induced podocytes injury through activation autophagy via inhibiting DUSP1 expression. IUBMB Life 72 (12), 2651–2662. 10.1002/iub.2393 33107695

[B28] Jeppesen D. K. Fenix A. M. Franklin J. L. Higginbotham J. N. Zhang Q. Zimmerman L. J. (2019). Reassessment of exosome composition. Cell 177 (2), 428–445. 10.1016/j.cell.2019.02.029 30951670 PMC6664447

[B29] Jeyaseelan K. Delić D. Eisele C. Schmid R. Baum P. Wiech F. (2016). Urinary exosomal miRNA signature in type II diabetic nephropathy patients. Plos One 11 (3), e0150154. 10.1371/journal.pone.0150154 26930277 PMC4773074

[B30] Ji J.-l. Shi H.-m. Li Z.-l. Jin R. Qu G.-t. Zheng H. (2023). Satellite cell-derived exosome-mediated delivery of microRNA-23a/27a/26a cluster ameliorates the renal tubulointerstitial fibrosis in mouse diabetic nephropathy. Acta Pharmacol. Sin. 44 (12), 2455–2468. 10.1038/s41401-023-01140-4 37596398 PMC10692096

[B31] Jia Y. Yu L. Ma T. Xu W. Qian H. Sun Y. (2022). Small extracellular vesicles isolation and separation: current techniques, pending questions and clinical applications. Theranostics 12 (15), 6548–6575. 10.7150/thno.74305 36185597 PMC9516236

[B32] Jiang Z.-z. Liu Y.-m. Niu X. Yin J.-y. Hu B. Guo S.-c. (2016). Exosomes secreted by human urine-derived stem cells could prevent kidney complications from type I diabetes in rats. Stem Cell Res. & Ther. 7 (1), 24. 10.1186/s13287-016-0287-2 26852014 PMC4744390

[B33] Johnson J. Law S. Q. K. Shojaee M. Hall A. S. Bhuiyan S. Lim M. B. L. (2023). First‐in‐human clinical trial of allogeneic, platelet‐derived extracellular vesicles as a potential therapeutic for delayed wound healing. J. Extracell. Vesicles 12 (7), e12332. 10.1002/jev2.12332 37353884 PMC10290200

[B34] Kalluri R. LeBleu V. S. (2020). The biology, function, and biomedical applications of exosomes. Science 367 (6478), eaau6977. 10.1126/science.aau6977 32029601 PMC7717626

[B35] Kanasaki K. Taduri G. Koya D. (2013). Diabetic nephropathy: the role of inflammation in fibroblast activation and kidney fibrosis. Front. Endocrinol. 4, 7. 10.3389/fendo.2013.00007 PMC3565176 23390421

[B36] Kanwar Y. S. Sun L. Xie P. Liu F.-y. Chen S. (2011). A glimpse of various pathogenetic mechanisms of diabetic nephropathy. Annu. Rev. Pathology Mech. Dis. 6 (1), 395–423. 10.1146/annurev.pathol.4.110807.092150 PMC3700379 21261520

[B37] Kim H. Bae Y.-U. Jeon J. S. Noh H. Park H. K. Byun D. W. (2019). The circulating exosomal microRNAs related to albuminuria in patients with diabetic nephropathy. J. Transl. Med. 17 (1), 236. 10.1186/s12967-019-1983-3 31331349 PMC6647278

[B38] Kwon H. Yang S. Lee J. Park B. Park K. Jung J. (2020). Combination treatment with human adipose tissue stem cell-derived exosomes and fractional CO2 laser for acne scars: a 12-week prospective, double-blind, randomized, split-face study. Acta Derm. Venereol. 100 (18), adv00310. 10.2340/00015555-3666 33073298 PMC9309822

[B39] Lee W.-C. Li L.-C. Ng H.-Y. Lin P.-T. Chiou T.T.-Y. Kuo W.-H. (2020). Urinary exosomal MicroRNA signatures in nephrotic, biopsy-proven diabetic nephropathy. J. Clin. Med. 9 (4), 1220. 10.3390/jcm9041220 32340338 PMC7231152

[B40] Li W. Yang S. Qiao R. Zhang J. (2018). Potential value of urinary exosome-derived let-7c-5p in the diagnosis and progression of type II diabetic nephropathy, Clin. Lab., 64, 709, 718. 10.7754/Clin.Lab.2018.171031 29739042

[B41] Li X. Lu L. Hou W. Huang T. Chen X. Qi J. (2021). Epigenetics in the pathogenesis of diabetic nephropathy. Acta Biochimica Biophysica Sinica 54 (2), 163–172. 10.3724/abbs.2021016 PMC9909329 35130617

[B42] Li B. Sun G. Yu H. Meng J. Wei F. (2022). Exosomal circTAOK1 contributes to diabetic kidney disease progression through regulating SMAD3 expression by sponging miR-520h. Int. Urology Nephrol. 54 (9), 2343–2354. 10.1007/s11255-022-03139-y 35142978

[B43] Li T. ci Liu T. Liu N. Zhang M. (2023). Changes in urinary exosomal protein CALM1 may serve as an early noninvasive biomarker for diagnosing diabetic kidney disease. Clin. Chim. Acta 547, 117466. 10.1016/j.cca.2023.117466 37406751

[B44] Li Y. Du Y. Liu Y. Chen X. Li X. Duan Y. (2024). Cardiomyocyte-derived small extracellular vesicle: a new mechanism driving diabetic cardiac fibrosis and cardiomyopathy. Theranostics 14 (15), 5926–5944. 10.7150/thno.99507 39346544 PMC11426245

[B45] Liang M. Zhu X. Zhang D. He W. Zhang J. Yuan S. (2022). Yi-Shen-Hua-Shi granules inhibit diabetic nephropathy by ameliorating podocyte injury induced by macrophage-derived exosomes. Front. Pharmacol. 13, 962606. 10.3389/fphar.2022.962606 36506555 PMC9732029

[B46] Ling L. Tan Z. Zhang C. Gui S. Cui Y. Hu Y. (2019). CircRNAs in exosomes from high glucose-treated glomerular endothelial cells activate mesangial cells. Am. J. Transl. Res. 11 (8), 4667–4682. 31497190 PMC6731409

[B47] Liu D. Liu F. Li Z. Pan S. Xie J. Zhao Z. (2021). HNRNPA1-mediated exosomal sorting of miR-483-5p out of renal tubular epithelial cells promotes the progression of diabetic nephropathy-induced renal interstitial fibrosis. Cell Death & Dis. 12 (3), 255. 10.1038/s41419-021-03460-x PMC7946926 33692334

[B48] Liu J.-L. Zhang L. Huang Y. Li X.-H. Liu Y.-F. Zhang S.-M. (2023a). Epsin1-mediated exosomal sorting of Dll4 modulates the tubular-macrophage crosstalk in diabetic nephropathy. Mol. Ther. 31 (5), 1451–1467. 10.1016/j.ymthe.2023.03.027 37016580 PMC10188907

[B49] Liu Y. Li X. Zhao M. Wu Y. Xu Y. Li X. (2023b). Macrophage-derived exosomes promote activation of NLRP3 inflammasome and autophagy deficiency of mesangial cells in diabetic nephropathy. Life Sci. 330, 121991. 10.1016/j.lfs.2023.121991 37524161

[B50] Luo X. Li R. Yan L.-J. (2015). Roles of pyruvate, NADH, and mitochondrial complex I in redox balance and imbalance in β cell function and dysfunction. J. Diabetes Res. 2015, 512618–512712. 10.1155/2015/512618 26568959 PMC4629043

[B51] Lv L.-L. Feng Y. Wu M. Wang B. Li Z.-L. Zhong X. (2019). Exosomal miRNA-19b-3p of tubular epithelial cells promotes M1 macrophage activation in kidney injury. Cell Death & Differ. 27 (1), 210–226. 10.1038/s41418-019-0349-y PMC7206053 31097789

[B52] Martelli F. Barutta F. Tricarico M. Corbelli A. Annaratone L. Pinach S. (2013). Urinary exosomal MicroRNAs in incipient diabetic nephropathy. PLoS ONE 8 (11), e73798. 10.1371/journal.pone.0073798 24223694 PMC3817183

[B53] Meldolesi J. (2018). Exosomes and ectosomes in intercellular communication. Curr. Biol. 28 (8), R435–R444. 10.1016/j.cub.2018.01.059 29689228

[B54] Meng W. He C. Hao Y. Wang L. Li L. Zhu G. (2020). Prospects and challenges of extracellular vesicle-based drug delivery system: considering cell source. Drug Deliv. 27 (1), 585–598. 10.1080/10717544.2020.1748758 32264719 PMC7178886

[B55] Mishra D. D. Sahoo B. Maurya P. K. Sharma R. Varughese S. Prasad N. (2023). Therapeutic potential of urine exosomes derived from rats with diabetic kidney disease. Front. Endocrinol. 14, 1157194. 10.3389/fendo.2023.1157194 PMC10213426 37251672

[B56] Munkonda M. N. Akbari S. Landry C. Sun S. Xiao F. Turner M. (2018). Podocyte‐derived microparticles promote proximal tubule fibrotic signaling via p38 MAPK and CD36. J. Extracell. Vesicles 7 (1), 1432206. 10.1080/20013078.2018.1432206 29435202 PMC5804677

[B57] Ning Y. Zhou X. Wang G. Zhang L. Wang J. (2024). Exosome miR-30a-5p regulates glomerular endothelial cells' EndMT and angiogenesis by modulating Notch1/VEGF signaling pathway and angiogenesis by modulating Notch1/VEGF signaling pathway. Curr. Gene Ther. 24(2)**,** 159–177. 10.2174/0115665232258527230919071328 37767799

[B58] Nunez Lopez Y. O. Casu A. Kovacova Z. Petrilli A. M. Sideleva O. Tharp W. G. (2022). Coordinated regulation of gene expression and microRNA changes in adipose tissue and circulating extracellular vesicles in response to pioglitazone treatment in humans with type 2 diabetes. Front. Endocrinol. 13, 955593. 10.3389/fendo.2022.955593 PMC9471675 36120427

[B59] Ogurtsova K. da Rocha Fernandes J. D. Huang Y. Linnenkamp U. Guariguata L. Cho N. H. (2017). IDF Diabetes Atlas: global estimates for the prevalence of diabetes for 2015 and 2040. Diabetes Res. Clin. Pract. 128, 40–50. 10.1016/j.diabres.2017.03.024 28437734

[B60] Ormazabal V. Nair S. Carrión F. McIntyre H. D. Salomon C. (2022). The link between gestational diabetes and cardiovascular diseases: potential role of extracellular vesicles. Cardiovasc. Diabetol. 21 (1), 174. 10.1186/s12933-022-01597-3 36057662 PMC9441052

[B61] Pan Y. Yang H. Chen T. Jin J. Ruan L. Hu L. (2022). Extracellular vesicles metabolic changes reveals plasma signature in stage-dependent diabetic kidney disease. Ren. Fail. 44 (1), 1840–1849. 10.1080/0886022x.2022.2118067 36368309 PMC9662026

[B62] Park G. H. Kwon H. H. Seok J. Yang S. H. Lee J. Park B. C. (2023). Efficacy of combined treatment with human adipose tissue stem cell-derived exosome-containing solution and microneedling for facial skin aging: a 12-week prospective, randomized, split-face study. J. Cosmet. Dermatol 22 (12), 3418–3426. 10.1111/jocd.15872 37377400

[B63] Patel D. B. Santoro M. Born L. J. Fisher J. P. Jay S. M. (2018). Towards rationally designed biomanufacturing of therapeutic extracellular vesicles: impact of the bioproduction microenvironment. Biotechnol. Adv. 36 (8), 2051–2059. 10.1016/j.biotechadv.2018.09.001 30218694 PMC6250573

[B64] Richards T. Patel H. Patel K. Schanne F. (2023). Endogenous lipid carriers-bench-to-bedside roadblocks in production and drug loading of exosomes. Pharm. (Basel) 16 (3), 421. 10.3390/ph16030421 PMC10058361 36986523

[B65] Sands J. M. Mohan A. Singh R. S. Kumari M. Garg D. Upadhyay A. (2016). Urinary exosomal microRNA-451-5p is a potential early biomarker of diabetic nephropathy in rats. Plos One 11 (4), e0154055. 10.1371/journal.pone.0154055 27101382 PMC4839711

[B66] Selby N. M. Taal M. W. (2020). An updated overview of diabetic nephropathy: diagnosis, prognosis, treatment goals and latest guidelines. Diabetes, Obes. Metabolism 22 (S1), 3–15. 10.1111/dom.14007 32267079

[B67] Shaban A. M. Raslan M. Qahl S. H. Elsayed K. Abdelhameed M. S. Oyouni A. A. A. (2022). Ameliorative effects of camel milk and its exosomes on diabetic nephropathy in rats. Membranes 12 (11), 1060. 10.3390/membranes12111060 36363614 PMC9697163

[B68] Sidhom K. Obi P. O. Saleem A. (2020). A review of exosomal isolation methods: is size exclusion chromatography the best option? Int. J. Mol. Sci. 21 (18), 6466. 10.3390/ijms21186466 32899828 PMC7556044

[B69] Singh A. Pore S. K. Bhattacharyya J. (2024). Encapsulation of telmisartan inside insulinoma-cell-derived extracellular vesicles outperformed biomimetic nanovesicles in modulating the pancreatic inflammatory microenvironment. J. Mater. Chem. B 12 (40), 10294–10308. 10.1039/d4tb00808a 39269191

[B70] Sinha N. Kumar V. Puri V. Nada R. Rastogi A. Jha V. (2020). Urinary exosomes: potential biomarkers for diabetic nephropathy. Nephrol. Carlt. 25 (12), 881–887. 10.1111/nep.13720 32323449

[B71] Sinha N. Puri V. Kumar V. Nada R. Rastogi A. Jha V. (2023). Urinary exosomal miRNA-663a shows variable expression in diabetic kidney disease patients with or without proteinuria. Sci. Rep. 13 (1), 4516. 10.1038/s41598-022-26558-4 36934129 PMC10024703

[B72] Srivastava S. P. Koya D. Kanasaki K. (2013). MicroRNAs in kidney fibrosis and diabetic nephropathy: roles on EMT and EndMT. BioMed Res. Int. 2013, 125469–125510. 10.1155/2013/125469 24089659 PMC3780472

[B73] Su H. Qiao J. Hu J. Li Y. Lin J. Yu Q. (2020). Podocyte-derived extracellular vesicles mediate renal proximal tubule cells dedifferentiation via microRNA-221 in diabetic nephropathy. Mol. Cell. Endocrinol. 518, 111034. 10.1016/j.mce.2020.111034 32926967

[B74] Sun Y. Zhao Y. Lu Y. Li H. Xiang J. Yang D. (2023). Urinary stem cell-derived exocrine circRNA ATG7 regulates the SOCS1/STAT3 signaling pathway through miR-4500, inhibits M1 macrophage polarization, and alleviates the progression of diabetes nephropathy. Int. Urology Nephrol. 56 (4), 1449–1463. 10.1007/s11255-023-03819-3 PMC10924005 37815664

[B75] Tang S. C. W. Yiu W. H. (2020). Innate immunity in diabetic kidney disease. Nat. Rev. Nephrol. 16 (4), 206–222. 10.1038/s41581-019-0234-4 31942046

[B76] Tang S. Leung J. C. K. Abe K. Chan K. W. Chan L. Y. Y. Chan T. M. (2003). Albumin stimulates interleukin-8 expression in proximal tubular epithelial cells *in vitro* and *in vivo* . J. Clin. Investigation 111 (4), 515–527. 10.1172/JCI16079 PMC151921 12588890

[B77] Tsai Y.-C. Hung W.-W. Chang W.-A. Wu P.-H. Wu L.-Y. Lee S.-C. (2021). Autocrine exosomal fibulin-1 as a target of MiR-1269b induces epithelial–mesenchymal transition in proximal tubule in diabetic nephropathy. Front. Cell Dev. Biol. 9, 789716. 10.3389/fcell.2021.789716 34977033 PMC8718747

[B78] Umanath K. Lewis J. B. (2018). Update on diabetic nephropathy: core curriculum 2018. Am. J. Kidney Dis. 71 (6), 884–895. 10.1053/j.ajkd.2017.10.026 29398179

[B79] Wang Y.-Y. Tang L.-Q. Wei W. (2018). Berberine attenuates podocytes injury caused by exosomes derived from high glucose-induced mesangial cells through TGFβ1-PI3K/AKT pathway. Eur. J. Pharmacol. 824, 185–192. 10.1016/j.ejphar.2018.01.034 29378192

[B80] Wang S. Bao L. Fu W. Deng L. Ran J. (2021). Protective effect of exosomes derived from bone marrow mesenchymal stem cells on rats with diabetic nephropathy and its possible mechanism. Am. J. Transl. Res. 13 (6), 6423–6430. 34306382 PMC8290711

[B81] Wang Z. Sun W. Li R. Liu Y. (2022). miRNA-93-5p in exosomes derived from M2 macrophages improves lipopolysaccharide-induced podocyte apoptosis by targeting Toll-like receptor 4. Bioengineered 13 (3), 7683–7696. 10.1080/21655979.2021.2023794 35291915 PMC9208503

[B82] Wang J. Tao Y. Zhao F. Liu T. Shen X. Zhou L. (2023). Expression of urinary exosomal miRNA-615-3p and miRNA-3147 in diabetic kidney disease and their association with inflammation and fibrosis. Ren. Fail. 45 (1), 2121929. 10.1080/0886022x.2022.2121929 36695327 PMC9879181

[B83] Welsh J. A. Goberdhan D. C. I. O'Driscoll L. Buzas E. I. Blenkiron C. Bussolati B. (2024). Minimal information for studies of extracellular vesicles (MISEV2023): from basic to advanced approaches. J. Extracell. Vesicles 13 (2), e12404. 10.1002/jev2.12404 38326288 PMC10850029

[B84] Wen J. Ma Z. Livingston M. J. Zhang W. Yuan Y. Guo C. (2020). Decreased secretion and profibrotic activity of tubular exosomes in diabetic kidney disease. Am. J. Physiology-Renal Physiology 319 (4), F664–F673. 10.1152/ajprenal.00292.2020 PMC7642884 32715764

[B85] Wu X. Gao Y. Xu L. Dang W. Yan H. Zou D. (2017). Exosomes from high glucose-treated glomerular endothelial cells trigger the epithelial-mesenchymal transition and dysfunction of podocytes. Sci. Rep. 7 (1), 9371. 10.1038/s41598-017-09907-6 28839221 PMC5571220

[B86] Xiang E. Han B. Zhang Q. Rao W. Wang Z. Chang C. (2020). Human umbilical cord-derived mesenchymal stem cells prevent the progression of early diabetic nephropathy through inhibiting inflammation and fibrosis. Stem Cell Res. & Ther. 11 (1), 336. 10.1186/s13287-020-01852-y 32746936 PMC7397631

[B87] Xie Y. Jia Y. Cuihua X. Hu F. Xue M. Xue Y. (2017). Urinary exosomal MicroRNA profiling in incipient type 2 diabetic kidney disease. J. Diabetes Res. 2017, 6978984–6979010. 10.1155/2017/6978984 29038788 PMC5605810

[B88] Yamazaki T. Mimura I. Tanaka T. Nangaku M. (2021). Treatment of diabetic kidney disease: current and future. Diabetes & Metabolism J. 45 (1), 11–26. 10.4093/dmj.2020.0217 PMC7850867 33508907

[B89] Yan L.-J. Jing S. Wu J. Luo X. (2016). Hyperglycemic stress and carbon stress in diabetic glucotoxicity. Aging Dis. 7 (1), 90–110. 10.14336/ad.2015.0702 26816666 PMC4723237

[B90] Yang J. Liu D. Liu Z. (2022). Integration of metabolomics and proteomics in exploring the endothelial dysfunction mechanism induced by serum exosomes from diabetic retinopathy and diabetic nephropathy patients. Front. Endocrinol. 13, 830466. 10.3389/fendo.2022.830466 PMC8991685 35399949

[B91] Yang H. Bai Y. Fu C. Liu W. Diao Z. (2023). Exosomes from high glucose-treated macrophages promote epithelial–mesenchymal transition of renal tubular epithelial cells via long non-coding RNAs. BMC Nephrol. 24 (1), 24. 10.1186/s12882-023-03065-w 36717805 PMC9887774

[B92] Zang J. Maxwell A. P. Simpson D. A. McKay G. J. (2019). Differential expression of urinary exosomal MicroRNAs miR-21-5p and miR-30b-5p in individuals with diabetic kidney disease. Sci. Rep. 9 (1), 10900. 10.1038/s41598-019-47504-x 31358876 PMC6662907

[B93] Zhang H. Liu J. Qu D. Wang L. Wong C. M. Lau C.-W. (2018). Serum exosomes mediate delivery of arginase 1 as a novel mechanism for endothelial dysfunction in diabetes. Proc. Natl. Acad. Sci. 115 (29), E6927–E6936. 10.1073/pnas.1721521115 29967177 PMC6055191

[B94] Zhang Y. Bi J. Huang J. Tang Y. Du S. Li P. (2020). Exosome: a review of its classification, isolation techniques, storage, diagnostic and targeted therapy applications. Int. J. Nanomedicine 15, 6917–6934. 10.2147/ijn.S264498 33061359 PMC7519827

[B95] Zhang Y. Le X. Zheng S. Zhang K. He J. Liu M. (2022). MicroRNA-146a-5p-modified human umbilical cord mesenchymal stem cells enhance protection against diabetic nephropathy in rats through facilitating M2 macrophage polarization. Stem Cell Res. & Ther. 13 (1), 171. 10.1186/s13287-022-02855-7 35477552 PMC9044847

[B96] Zhang M. Hu S. Liu L. Dang P. Liu Y. Sun Z. (2023). Engineered exosomes from different sources for cancer-targeted therapy. Signal Transduct. Target. Ther. 8 (1), 124. 10.1038/s41392-023-01382-y 36922504 PMC10017761

[B97] Zhang K. Zheng S. Wu J. He J. Ouyang Y. Ao C. (2024). Human umbilical cord mesenchymal stem cell‐derived exosomes ameliorate renal fibrosis in diabetic nephropathy by targeting Hedgehog/SMO signaling. FASEB J. 38 (7), e23599. 10.1096/fj.202302324R 38572590

[B98] Zhao Y. Shen A. Guo F. Song Y. Jing N. Ding X. (2020). Urinary exosomal MiRNA-4534 as a novel diagnostic biomarker for diabetic kidney disease. Front. Endocrinol. 11, 590. 10.3389/fendo.2020.00590 PMC7484971 32982978

[B99] Zhou F. Huang L. Qu S. L. Chao R. Yang C. Jiang Z. S. (2019). The emerging roles of extracellular vesicles in diabetes and diabetic complications. Clin. Chim. Acta 497, 130–136. 10.1016/j.cca.2019.07.032 31361990

[B100] Zhou S. Fang J. Hu M. Pan S. Liu D. Xing G. (2021). Determining the influence of high glucose on exosomal lncRNAs, mRNAs, circRNAs and miRNAs derived from human renal tubular epithelial cells. Aging (Albany NY) 13 (6), 8467–8480. 10.18632/aging.202656 33714195 PMC8034913

[B101] Zhou M. Li Y. J. Tang Y. C. Hao X. Y. Xu W. J. Xiang D. X. (2022). Apoptotic bodies for advanced drug delivery and therapy. J. Control Release 351, 394–406. 10.1016/j.jconrel.2022.09.045 36167267

[B102] Zhu M. Sun X. Qi X. Xia L. Wu Y. (2020). Exosomes from high glucose-treated macrophages activate macrophages and induce inflammatory responses via NF-κB signaling pathway *in vitro* and *in vivo* . Int. Immunopharmacol. 84, 106551. 10.1016/j.intimp.2020.106551 32388490

[B103] Zhu Y. Zha F. Tang B. Ji T. T. Li X. Y. Feng L. (2021). Exosomal hsa_circ_0125310 promotes cell proliferation and fibrosis in diabetic nephropathy via sponging miR‐422a and targeting the IGF1R/p38 axis. J. Cell. Mol. Med. 26 (1), 151–162. 10.1111/jcmm.17065 34854210 PMC8742240

